# The efficacy of platelet-rich plasma preparation protocols in the treatment of osteoarthritis: a network meta-analysis of randomized controlled trials

**DOI:** 10.1186/s13018-025-06026-1

**Published:** 2025-06-24

**Authors:** Dongsheng Yu, Jiani Zhao, Kun Zhao

**Affiliations:** 1 Center for Rehabilitation Medicine, Department of Orthopedics, Zhejiang Provincial People’s Hospital (Affiliated People’s Hospital), Hangzhou Medical College, Hangzhou, Zhejiang, China; 2 School of Public Health, Hangzhou Medical College, Hangzhou, China; 3 Center for Rehabilitation Medicine, Rehabilitation & Sports Medicine Research Institute of Zhejiang Province, Department of Rehabilitation Medicine, Zhejiang Provincial People’s Hospital (Affiliated People’s Hospital), Hangzhou Medical College, Hangzhou, Zhejiang China

**Keywords:** Platelet rich plasma, Hyaluronic acid, Saline, Cartilage, Osteoarthritis, Knee, Meta-analysis, Systematic review

## Abstract

**Purpose:**

Osteoarthritis (OA) is a widespread joint disease characterized by the gradual loss of cartilage. Intra-articular injections, including platelet-rich plasma (PRP), are commonly used for treatment, but the optimal PRP preparation method remains debated. This study aims to perform a network meta-analysis of randomized controlled trials to compare the efficacy of different PRP preparation methods and determine the most effective protocols.

**Methods:**

The literature search was conducted based on PRISMA guidelines. Randomized controlled trials (RCTs) evaluating intra-articular injectables in osteoarthritic knees were included. Data were extracted, and the Western Ontario and McMaster Universities Osteoarthritis Index (WOMAC) scores were analyzed at 3, 6, and 12–18 months. Clinical outcomes were compared using a frequentist network meta-analysis, and treatment options were ranked using the P-Score. Statistical analysis was performed using R 4.3.2.

**Results:**

Twenty-three RCTs with 1752 patients were included. Treatments included PRP, plasma rich in growth factor (PRGF), leukocyte-poor PRP (LP-PRP), leukocyte-rich PRP (LR-PRP), hyaluronic acid (HA), and saline placebo. Leukocyte-rich PRP with low platelet concentration increase, using both anticoagulant and activator showed the best effects on WOMAC pain and stiffness scores within 6 months (WMD = 26.02; 95% CrI, 0.92–52.46). Leukocyte-poor PRP with high platelet concentration increase, using anticoagulant without activator was most effective for WOMAC function and stiffness at 12–18 months (WMD = 18.94; 95% CrI, 8.34–28.12). Long-term results indicated Leukocyte-poor PRP with low platelet concentration increase, using anticoagulant without activator yielded the best outcomes for cartilage repair and function (WMD = 17.09; 95% CrI, -8.4 to 42.78).

**Conclusions:**

Optimizing OA treatment involves tailoring PRP protocols to disease stage, with low platelet, high leukocyte PRP (RPRP_LPC_Y_Y) recommended for early OA due to its anti-inflammatory effects and high platelet, low leukocyte PRP (PPRP-HPC) preferred for advanced OA to promote tissue repair and regeneration.

**Supplementary Information:**

The online version contains supplementary material available at 10.1186/s13018-025-06026-1.

## Introduction

Osteoarthritis (OA) affects over 520 million people worldwide, often progressing to total knee arthroplasty (TKA) in advanced stages [38, 60]. Intra-articular injections remain a key conservative treatment, providing pain relief and functional improvement by delivering therapeutic agents directly into the joint [6, 37, 42, 56].

Platelet-rich plasma (PRP) is widely used in early-stage OA due to its anti-inflammatory effects and ability to promote tissue regeneration [4, 29, 53]. Leukocyte-rich PRP (LR-PRP) can enhance acute-phase inflammation, aiding tissue repair, while its platelet concentration influences growth factor release, affecting chondrocyte activity and angiogenesis [8, 13, 35, 36, 52, 54, 58, 59]. However, excessive platelet levels may impair tissue repair27. Activators promote rapid growth factor release [49]and when used with anticoagulants, they may enhance platelet activation11. These variations in PRP composition significantly impact clinical outcomes.

Different PRP preparation methods result in compositional and therapeutic discrepancies. Magalon et al. compared five protocols and found significant differences in platelet and growth factor levels, highlighting the need for standardization [36]. Despite this, no meta-analysis has systematically compared PRP preparation methods in knee OA. The PAW classification system, which considers platelet concentration, activation status, and leukocyte content, provides a framework for standardization [15]. Based on this system, we analyzed four PRP preparation methods to evaluate their short- and long-term effects and determine optimal protocols for different OA stages.

## Methods

### Study selection

Two independent reviewers conducted the literature search following the PRISMA (Preferred Reporting Items for Systematic Reviews and Meta-Analyses) guidelines [27]. Discrepancies were resolved through reconciliation by a third author. The initial screening involved evaluating titles and abstracts, with potentially relevant studies undergoing a full-text review. Additionally, the references of all included studies were manually screened to identify any additional articles meeting the inclusion criteria.

### Search strategy

The search strategy was conducted across PubMed, EMBASE, and the Cochrane Library databases on October 16, 2023. Keywords and controlled vocabulary related to osteoarthritis (e.g., “osteoarthr*” or “degenerative arthritis”) and platelet-rich plasma (PRP) (e.g., “PRP,” “buffy layer,” or “platelet gel”) were used, combined with filters for human studies and clinical trial designs such as randomized controlled trials. Boolean operators and field-specific queries refined the search, ensuring comprehensive retrieval of relevant literature. The search strategy is presented in Appendix 1. The combined strategy yielded 519 records from PubMed, 764 from the Cochrane Library, and 1,108 from EMBASE. No restrictions were placed on publication dates.

### Eligibility criteria

The inclusion criteria were: (1) randomized controlled trial comparing intra-articular injections in the knee, (2) published in a peer-reviewed journal, (3) published in English, (4) includes outcome scores such as WOMAC, KOOS, ROM, VAS, KC, MAA, IKDC, and Tegner, and (5) full text of studies available.The exclusion criteria were the following: (1) studies without patient-reported outcome measures (e.g., WOMAC, VAS, IKDC); (2) studies with incomplete or non-convertible outcome data; (3) duplicate publications based on the same cohort; (4) conference abstracts, reviews, or editorials; (5) basic science or preclinical research (e.g., animal studies, in vitro studies).

### Data extraction

Data extraction was carried out by two independent reviewers using a standardized data sheet. Information collected included study characteristics (study title, country, diagnostic criteria, treatment arms, patient follow-up duration, Kellgren-Lawrence grade, symptom duration, patient age, percentage of female participants, and BMI). Details of PRP application were also gathered, such as injection method, frequency, volume, and site. For PRP preparation methods, the type of anticoagulant used, whether an activator was added, and the specifics of the preparation system (number of spins, speed, and equipment) were noted. Additionally, the leukocyte content and mean-fold change in platelet concentration were recorded. The analysis encompassed a summary of study characteristics and findings, highlighting variability in PRP preparation and protocols. Meta-analyses were performed where data were homogeneous, using a random-effects model, with heterogeneity assessed via the I² statistic. A network meta-analysis (NMA) allowed for both direct and indirect comparisons of different PRP preparation methods. Sensitivity analyses were conducted to ensure robustness, and publication bias was assessed with funnel plots and Egger’s test.

### Data analysis

Based on the concentration of leukocytes in PRP (categorized into leukocyte-poor PRP and leukocyte-rich PRP) and the degree of platelet concentration increase (low increase < 3-fold, moderate increase 3-fold to < 5-fold, high increase ≥ 5-fold), and the usage of activators and anticoagulants(Used: Y, Not Used: N, Unknown: X), the preparation methods were classified into 14 categories in Table 1.

**Table 1:** PRP Preparation classification by leukocyte, platelet concentration, and additives

	Abbreviation	Full Name
1	RPRP_HPC_Y_Y	Leukocyte-rich PRP with high platelet concentration increase, using both anticoagulant and activator
2	RPRP_HPC_Y_N	Leukocyte-rich PRP with high platelet concentration increase, using anticoagulant without activator
3	RPRP_HPC_X_N	Leukocyte-rich PRP with high platelet concentration increase, using anticoagulant with unknown activator status
4	RPRP_MPC_Y_Y	Leukocyte-rich PRP with moderate platelet concentration increase, using both anticoagulant and activator
5	RPRP_MPC_Y_N	Leukocyte-rich PRP with moderate platelet concentration increase, using anticoagulant without activator
6	RPRP_LPC_Y_Y	Leukocyte-rich PRP with low platelet concentration increase, using both anticoagulant and activator
7	PPRP_LPC_Y_N	Leukocyte-poor PRP with low platelet concentration increase, using anticoagulant without activator
8	PPRP_HPC_Y_N	Leukocyte-poor PRP with high platelet concentration increase, using anticoagulant without activator
9	PPRP_MPC_Y_Y	Leukocyte-poor PRP with moderate platelet concentration increase, using both anticoagulant and activator
10	PPRP_MPC_Y_N	Leukocyte-poor PRP with moderate platelet concentration increase, using anticoagulant without activator
11	PPRP_MPC_X_Y	Leukocyte-poor PRP with moderate platelet concentration increase, using anticoagulant and activator
12	PPRP_LPC_Y_Y	Leukocyte-poor PRP with low platelet concentration increase, using both anticoagulant and activator
13	PPRP_LPC_N_N	Leukocyte-poor PRP with low platelet concentration increase, without anticoagulant and activator
14	PPRP_LPC_X_N	Leukocyte-poor PRP with low platelet concentration increase, without anticoagulant and with unknown activator status

The assessment of clinical outcomes included WOMAC pain sub score, WOMAC function sub scores WOMAC stiffness sub scores, WOMAC total scores. The results were evaluated at 6 and 12–18 months or the closest reported follow-up times within 3 months. WOMAC pain sub scores, WOMAC function sub scores WOMAC stiffness sub scores, WOMAC total scores were normalized on a scale from 0 to 100.

### Statistical analysis

All statistical analyses were performed using R(version 4.3.2; R Foundation for Statistical Computing, Vienna, Austria). A frequentist approach to network meta-analysis with a random effects model was performed using the netmeta package version 0.9–6 in R [48]. For continuous outcomes, the relative effect sizes were reported as standardized mean differences (MD), and for dichotomous outcomes, the relative effect sizes were.

reported as odds ratios (OR). The effect sizes were reported with 95% confidence intervals (95% CI).Heterogeneity was quantitatively assessed using the I² statistic, supplemented by Cochran’s Q test to determine the presence of statistically significant differences. An I² > 50% was interpreted as moderate or higher heterogeneity, while an I² > 75% indicated substantial heterogeneity [25]. To explore the potential impact of study-level covariates and assess the stability of our findings, meta-regression analyses were conducted using key variables including mean age, mean body mass index (BMI), proportion of female participants, and total sample size. Given the heterogeneity in follow-up durations across included trials, outcome measures were stratified by three predefined timeframes: short-term (within 3 months), mid-term (6 months), and long-term (12 to 18 months), to reduce the impact of temporal variation on effect estimates. Publication bias was examined through visual inspection of funnel plots and tested quantitatively using Egger’s regression test. If asymmetry was identified or Egger’s test indicated significance (*P* < 0.05), we further applied trim-and-fill or non-parametric methods to evaluate the stability of the results. In addition, key methodological features of the included studies, such as comparator types (e.g., hyaluronic acid or saline), follow-up duration, and baseline demographics (age, sex, BMI), were carefully documented and considered in subgroup and sensitivity analyses to account for potential sources of bias. The frequentist analogue to the surface under the cumulative ranking (SUCRA) probabilities called the P-score was used to rank the treatments. This method allows each treatment to be ranked on a scale from 0 to 1, where 0 indicates the least effective treatment and 1 indicates the most effective [48].

## Results

### Search result

**Fig. 1:**
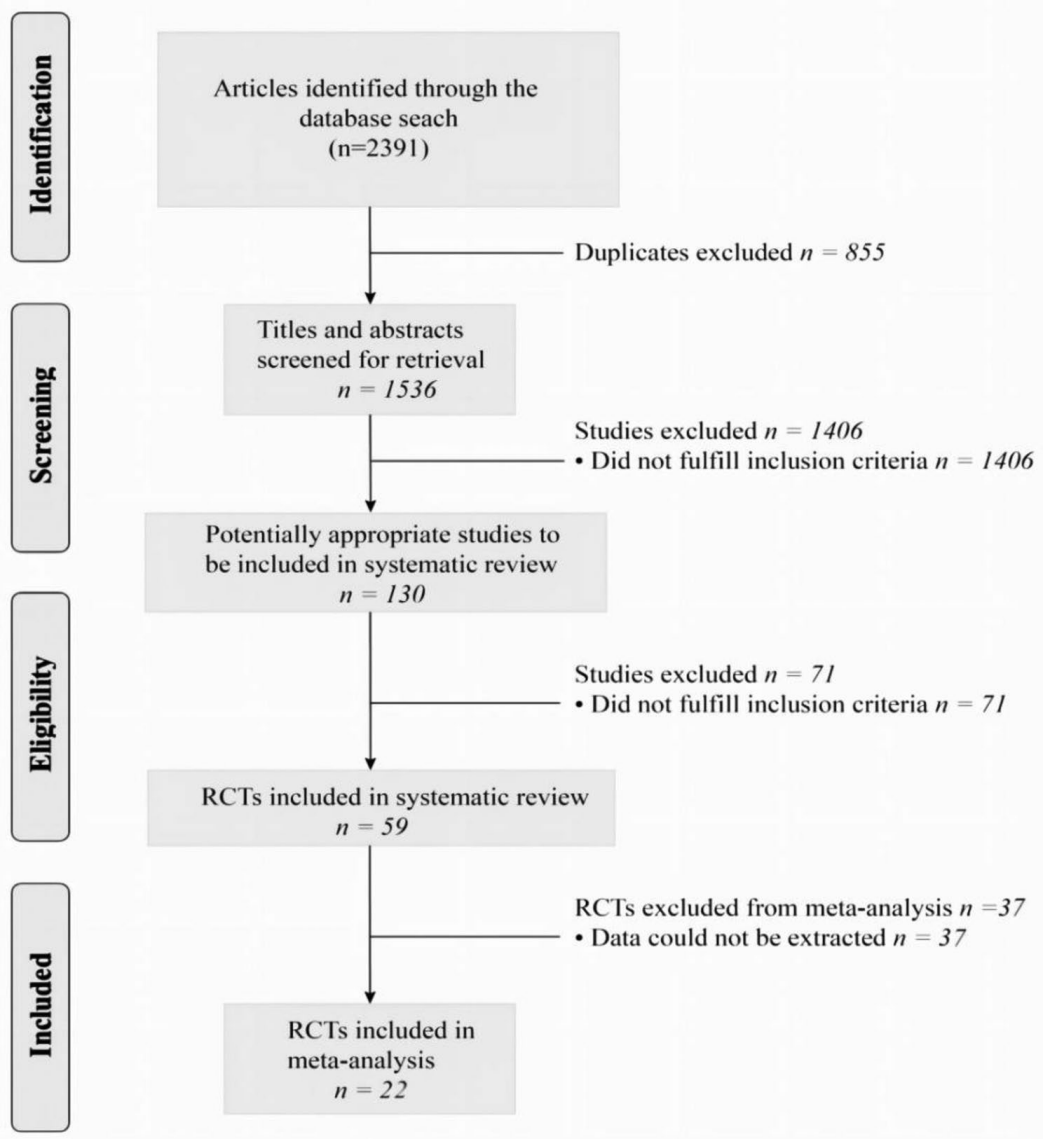
Flow diagram of the study selection procedure

The initial search resulted in 2391 papers. Of them, 855 were duplicates and, thus, excluded. Only 59 were RCTs. 37 of these were excluded because they did not match the topic or did not report quantitative data according to our outcomes of interest. T. Ultimately, 22 articles that met the eligibility criteria were included (Fig. 1) [6, 7, 12, 18–20, 23, 26, 30, 33, 34, 40, 42–46, 50, 51, 55, 61].A total of 1,752 patients, including 1,239 females, were enrolled in the study. The mean age of the patients included in the studies ranged from 50 to 75 years. The severity of the disease was primarily concentrated at Kellgren-Lawrence grades of 2 to 3. Details of the study characteristics are presented in Table 2, and network diagrams of available comparisons and studies are illustrated in Appendix 2.

**Table 2:** Characteristics of studies included in the analysis

Study	Level of evidence	K-L Grading	Arms	Patients Follow-up	Age (year)	Female (%)	BMI, kg/m2	PRP Operation Methods				PRP Preparation Methods		Outcome	measurement timepoint (month)
								Injection Volume (ml)	Anticoagulant	Activator Added	Preparation System (Times)	White Blood Cells	Platelet Mean-fold Change		
Su et al.,2018	**I**	2–3	PRP	25	54.16 ± 6.56	56.00%	28.17 ± 1.43	6	Y	Y	2	rich	5.61x	VAS, WOMAC	1,3,6,12,18
			HA	30	53.13 ± 6.41	60.00%	28.69 ± 1.13								
Tucker et al.,2021	**I**	2–3	PRP	11	57.5 ± 1.8	66.67%	30.9 ± 1.5	5	Y	Y	NR	rich	2.59x ± 0.68	VAS, WOMAC	3,6,12
			saline	6	57.2 ± 3.9	27.27%	29.1 ± 2.1								
Yaradilmis et al.,2020	**I**	2–3	three doses of LP-PRP	30	58.93 ± 6.25	90.00%	32.53 ± 6.25	3	Y	N	2	poor	4.6x	VAS, WOMAC	2,6,12
			three doses of LR-PRP	30	60.3 ± 7.65	86.70%	31.27 ± 4.08	3	Y	N	2	rich	1.9x		
			HA	30	63 ± 9.17	86.7%	32.4 ± 4.2								
Yurtbay et al.,2022	**I**	1–3	one dose of PRP	62	53.29 ± 12.97	33.87%	31.09 ± 5.52	5	Y	Y	1	rich	> 5x	KOOS, VAS	1,3,6,12,24
			three doses of LR-PRP	63	57.38 ± 8.78	14.29%	30.68 ± 4.63	5	Y	Y	1	rich	> 5x		
			one dose of NS	59	56.29 ± 10.53	18.64%	30.67 ± 4.51								
			three doses of NS	53	53.47 ± 11.31	33.96%	29.22 ± 4.79								
Lin et al.,2019	**I**	NR	PRP	31	61.17 ± 13.08	70.97%	23.98 ± 2.62	2	N	N	1	poor	1.81x ± 0.34	WOMAC, IKDC	1,2,6,12
			HA	29	62.53 ± 9.9	65.52%	26.26 ± 2.99								
			saline	27	62.23 ± 11.71	62.96%	24.98 ± 3.12								
Spaková et al.,2012	**I**	1–3	PRP	60	52.80 ± 12.43	45.00%	27.9 ± 4.1	3	Y	N	3	rich	4.5x	WOMAC	3,6
			HA	60	53.20 ± 14.53	48.33%	28.3 ± 4.0								
Himanshu et al.,2021	**I**	1–3	PRP	64	64.4	39.06%	24.90	8	Y	N	2	poor	6.25x	WOMAC, IKDC	1,3,6,12
			HA	68	65.8	38.24%	25.29								
Buendía-López et al.,2018	**I**	1–2	PRP	33	56.15 ± 3.001	51.52%	24.9 ± 0.32	5	NR	Y	2	poor	3.87x	WOMAC, VAS	6,12
			HA	32	56.63 ± 2.9	53.13%	24.9 ± 0.41								
Dório et al.,2021	**I**	2–3	PRP	20	66.4 ± 5.6	95.00%	28.3 ± 4.1	1.4-5	Y	N	2	poor	3x	WOMAC, KOOS	3,6
			saline	21	62.5 ± 8.1	90.48%	27.6 ± 3.8								
Duymus et al.,2017	**I**	2–3	PRP	33	60.4 ± 5.1	96.97%	27.6 ± 4.6	5	NR	N	1	rich	9-13x	WOMAC, VAS	1,3,6,12
			HA	34	60.3 ± 9.1	97.06%	28.4 ± 3.6								
Filardo et al.,2015	**I**	NR	PRP	94	53.32 ± 13.2	36.17%	26.6 ± 4.0	5	NR	Y	2	rich	4.6x ± 1.4	IKDC, KOOS	2,6,12
			HA	89	57.55 ± 11.8	41.57%	26.9 ± 4.4								
Huang et al.,2019	**I**	NR	PRP	40	54.5 ± 1.2	79.20%	25.23 ± 4.15	4	NT	N	1	poor	2x	WOMAC, VAS	3,6,12
			HA	40	54.8 ± 1.1	84.20%	24.51 ± 3.09								
Nunes-Tamashiro et al.,2022	**I**	2–3	PRP	34	67.6 ± 7.4	90.90%	29.22 ± 3.2	NR	Y	N	1	poor	2.5-5x	WOMAC, VAS	1,2,6,12
			saline	33	68 ± 6.2	88.20%	30.23 ± 4.1								
Lana et al.,2016	**I**	1–3	PRP	36	60.9 ± 7	80.60%	27.42 ± 6.89	5	Y	Y	2	rich	6.5x	WOMAC, VAS	1,3,6,12
			HA	36	60 ± 6.6	91.70%	28.24 ± 8.77								
Louis et al.,2018	**I**	NR	PRP	24	53.2 ± 11.7	41.67%	25.6 ± 2.9	3	Y	Y	2	rich	3.3x ± 0.7	WOMAC	1,3,6
			HA	24	48.5 ± 11.5	54.17%	27.0 ± 2.9								
Park et al.,2021	**I**	1–3	PRP	55	60.6 ± 8.2	70.91%	25.5 ± 2.2	3	Y	Y	1	rich	3x	IKDC, VAS, WOMAC	3,6
			HA	55	62.3 ± 9.6	85.45%	25.9 ± 2.8								
Patel et al.,2013	**I**	NR	PRP	27	53.11 ± 11.55	59.26%	26.28 ± 3.23	8	Y	Y	1	poor	3x	WOMAC, VAS	3,6
			saline	23	53.65 ± 8.17	73.91%	26.21 ± 2.93								
Hüseyin et al.,2019	**I**	1–3	PRP	30	61.30 ± 7.91	96.67%	30.37 ± 4.47	4	Y	Y	2	rich	4-6x	WOMAC, VAS	1,6
			saline	27	60.19 ± 6.80	88.89%	30.70 ± 3.97								
Raeissadat et al.,2017	**I**	2–3	PRGF	36	57.0 ± 7.18	80.56%	28.6 ± 2.82	5	Y	Y	3	poor	0	WOMAC, VAS	2,6
			HA	33	59.5 ± 7.54	81.82%	27.5 ± 2.9								
Raeissadat et al.,2020	**I**	2–3	PRGF	50	57.08 ± 7.3	72.00%	27.92 ± 2.7	5	Y	Y	3	poor	0	WOMAC, VAS	2,6,12
			HA	52	58.63 ± 7.09	71.15%	28.65 ± 3.02								
Raeissadat et al.,2021	**I**	2–3	PRP	52	56.09 ± 6.0	75.00%	27.41 ± 2.6	NR	Y	N	2	rich	4-6x	WOMAC, VAS	2,6,12
			PRGF	51	56.07 ± 6.3	72.55%	27.50 ± 2.1	NR	Y	Y	3	rich	5x		
			HA	49	57.91 ± 6.7	75.51%	27.46 ± 2.2								
Di Martino et al.,2022	**I**	1–3	LR-PRP	90	55.2 ± 9.8	31.11%	26.1 ± 4.5	5	Y	Y	2	rich	4.6x	IKDC, KOOS	2,6,12
			LP-PRP	85	55.7 ± 10.7	41.18%	27.4 ± 4.1	5	Y	Y	2	poor	4.4x		

According to the Cochrane risk-of-bias tool for assessing the overall risk of bias, 17 studies were rated as low risk, 6 studies 18,20,26,30,50,51 as moderate risk. Among them, 6 studies 18,20,26,30,50,51 did not specify the randomized scheme in the article. The results of the Cochrane risk-of-bias assessment are presented in Appendix 3.

### WOMAC pain

In the short-term, RPRP_LPC_Y_Y demonstrated the greatest efficacy compared to HA and Saline (WMD = 26.42; 95% CrI, 4.08 to 47.7), with the highest probability of being the best treatment (SUCRA = 0.98). The model showed minimal heterogeneity (global I² = 0%) and good fit (posterior mean residual deviance: 25.41/27).

For medium-term outcomes, RPRP_LPC_Y_Y remained the top-ranked treatment (SUCRA = 0.89), but its effect was less pronounced (WMD = 20.74; 95% CrI, -10.31 to 48.58). Long-term analysis was conducted in two sub-networks due to connectivity limitations: PPRP_HPC_Y_N had the most significant impact in the HA network (WMD = -1.71; 95% CrI, -4.41 to 0.98, SUCRA = 0.70), while RPRP_LPC_Y_Y was most effective in the Saline network (WMD = 5.61; 95% CrI, -16.93 to 27.88, SUCRA = 0.65).

### WOMAC function

Short-term analysis showed that RPRP_LPC_Y_Y was the most effective treatment (WMD = 18.98; 95% CrI, -4.47 to 42.73, SUCRA = 0.92). In the medium term, PPRP_HPC_Y_N demonstrated superior efficacy (WMD = 18.94; 95% CrI, 8.34 to 28.12, SUCRA = 0.94). Long-term results indicated that PPRP_LPC_Y_N was the highest-ranked treatment (WMD = 17.09; 95% CrI, -8.4 to 42.78, SUCRA = 0.86). All models showed good fit with minimal heterogeneity.

### WOMAC stiffness

RPRP_LPC_Y_Y was the most effective short-term intervention (WMD = 26.02; 95% CrI, 0.92 to 52.46, SUCRA = 0.98). In the medium term, PPRP_HPC_Y_N ranked highest (WMD = 3.52; 95% CrI, 1.22 to 5.96, SUCRA = 0.89). Long-term analysis was conducted in two sub-networks: PPRP_HPC_X_N was the top treatment in the HA network (WMD = -0.8; 95% CrI, -1.98 to 0.39, SUCRA = 0.77), while PPRP_MPC_Y_N ranked highest in the Saline network (WMD = 1.62; 95% CrI, -5.35 to 8.54, SUCRA = 0.73).

These results indicate that PRP preparations with high platelet and leukocyte concentrations, particularly RPRP_LPC_Y_Y and PPRP_HPC_Y_N, provide superior short- and medium-term benefits, while different PRP formulations maintain efficacy in the long term. The full network meta-analysis results are available in Appendix 4.

### Additional analysis

**Fig. 2:**
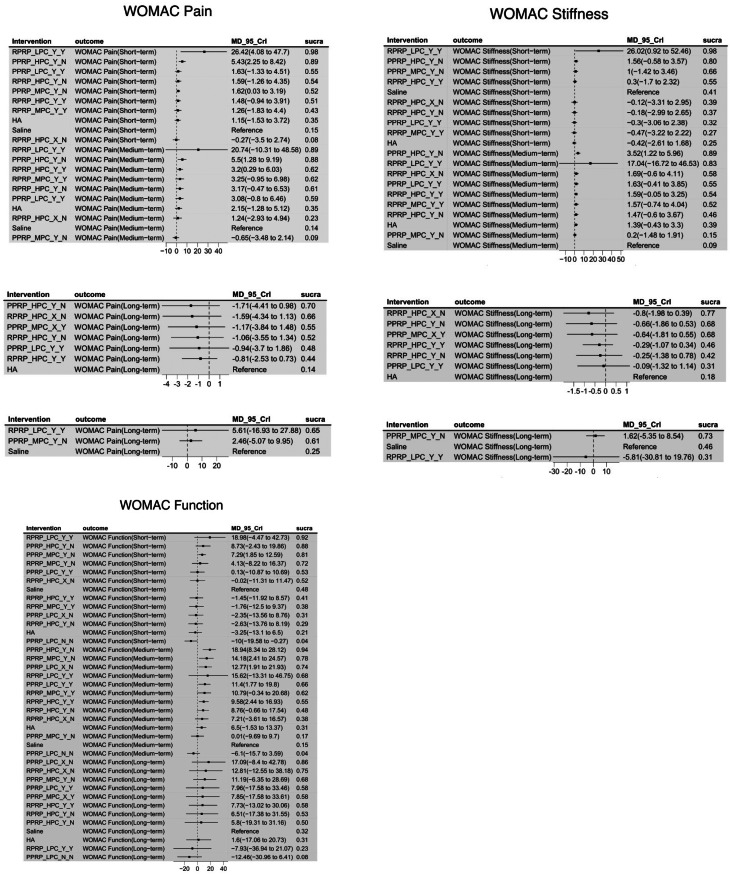
The forest plot illustrates the differences in WOMAC Pain, WOMAC Function, and WOMAC Stiffness scores in comparison to HA and Saline, along with the SCURA values

All the models had a good fit, and the potential scale reduction factor was very close to 1. No significant inconsistency was detected between other direct and indirect comparisons (*P* > 0.05) (Fig. 2).Network meta-regression showed no significant interactions between female, BMI, patient age, sample size, and treatment effect(Appendix 5).

## Discussion

The NMA evaluates the relative clinical efficacy of different PRP preparation protocols for osteoarthritis in comparison to standard treatments such as HA and saline.

The results revealed demonstrate that the effects of different PRP preparation methods on WOMAC Pain, WOMAC Function, and WOMAC Stiffness scores vary across in short-term, medium-term and long-term periods of OA treatment.

In the short-term follow-up, the RPRP_LPC_Y_Y preparation obtained best results in all WOMAC Pain, Function, and Stiffness scores, indicating the rich leukocyte and low platelet combination PRP with an immediate clinical efficacy in OA treatment. The inflammatory environment in OA pathology will activate local immune response and promote pro-inflammatory factors, which may further trigger and exacerbate the inflammatory reaction. These cytokines take an important role OA progress, as well as the development of pain in OA patient. For instance, TNF-α as a well-known inflammatory cytokine will stimulate nociceptors in OA patients when elevated in the synovium or joint fluid, which finally causes the sensation of OA-related pain [5]. Moreover, IL-1Ra as an anti-inflammatory mediator in OA, was found to have a higher expression in PRP with rich leukocyte than that with a lower leukocyte level [57]. Hence, as the increased leukocytes of RPRP_LPC_Y_Y preparation were injected into the joint, they might play a similar role with NSAIDs drugs in directly suppressing local inflammatory responses [10]. Early intervention to inflammatory environment can further reduce the production of pro-inflammatory cytokines and proteolytic enzymes associating with cellular catabolism and apoptosis [28, 62]. That may ultimately alleviate pain sensitization and tissue fibrosis, as well as prevent joint adhesions [47]. Moreover, well controlled pain allows OA patients to move their knee joints, resulting in joint stiffness relief and function improvement. Therefore, inhibition of inflammatory environment by leukocyte-rich, low-concentration platelet PRP alleviated pains and joint dysfunction of OA patients in an early point and then obtained remarkable WOMAC scores in the short-term follow-up.

PPRP_HPC preparations manifested the most favorable outcomes in the long-term follow-up suggesting that the combined therapy of leukocyte-poor and high-platelet PRP holds significant clinical efficacy in the treatment of osteoarthritis. The low-leukocyte and high-platelet PRP combination enhanced the secretion of growth factors which may play a crucial role in cartilage repair and functional recovery in osteoarthritis [35]. For instance, PDGF and TGF-βcontained in the PRP can stimulate chondrocytes proliferation and differentiation contributing to the repair of cartilage and other joint tissues [31, 36]. The enhanced secretion of IGF-1 can promotes collagen production and extracellular matrix production promoting tendon and cartilage healing [41]. These growth factors also enhance the blood supply around the joint, which may further accelerate cartilage regeneration and ultimately improve joint function [21, 22]. Beyond these effects, high-platelet PRP induces the temporal and spatial release of bioactive molecules that not only stimulate resident chondrocytes but also attract mesenchymal stem cells to the injured site, fostering a regenerative microenvironment [1, 2]. The sustained delivery of factors such as VEGF and IGF-1 contributes to subchondral vascular remodeling and matrix biosynthesis [2]while the modulation of the local immune response—characterized by downregulation of catabolic cytokines like IL-1β and TNF-α and upregulation of anti-inflammatory mediators like IL-10—helps to mitigate chronic inflammation and create favorable conditions for tissue repair [1, 2]. In certain patient populations, individual variability in regenerative capacity and immune status may influence response to PRP therapy, making the optimization of leukocyte and platelet profiles particularly important for enhancing clinical outcomes [3]. It is also important to note that the efficacy of PRP may be affected by concurrent medication use; agents such as NSAIDs or corticosteroids have been shown to impair platelet activation and suppress growth factor release, thereby potentially attenuating the therapeutic effect of the injection [24]. While cartilage repair is not an instant process, PPRP_HPC preparations presented no significant advantages in the early stage of functional scores. PPRP_HPC gradually showed benefits in supporting tissue regeneration over time which is reflected in better long-term WOMAC scores [22]. Thus, in the mid-term follow-up (6–12 months), dual roles of different leukocyte and platelet concentrations might cause confused statuses of inflammation control and tissue regeneration in the OA joint and lead to varied clinical outcomes.

### Limitations

This study has several important limitations. First, the presence of clinical and methodological heterogeneity among the included studies — such as variations in patient characteristics, PRP preparation protocols, injection frequencies, control interventions (HA or saline), and outcome assessment tools — reduces direct comparability and may impact the reliability of pooled estimates. To explore potential sources of heterogeneity, meta-regression analyses were performed based on study-level variables, including mean age, BMI, sex distribution, and total sample size. Second, the lack of standardized classification criteria for PRP preparations across studies undermines the consistency and scientific reproducibility of comparisons. Third, although publication bias was assessed using funnel plots and Egger’s test, the presence of asymmetry in some comparisons may still influence the robustness of findings. Fourth, the scarcity of long-term follow-up data, particularly for trials using HA or saline controls, limits the ability to draw definitive conclusions about sustained treatment efficacy. Lastly, six studies were rated as having a moderate risk of bias, which could influence the overall quality and credibility of the findings. These limitations should be considered when interpreting the results and highlight key areas for methodological improvement in future research.

## Conclusions

This network meta-analysis supports a tailored approach to PRP treatment based on osteoarthritis severity and therapeutic objectives. For early-stage OA, where symptoms are primarily pain and stiffness, low platelet concentration, high leukocyte PRP (e.g., RPRP_LPC_Y_Y) appears more effective, potentially due to its anti-inflammatory properties. In contrast, for advanced OA with significant cartilage damage, high platelet concentration, low leukocyte PRP (e.g., PPRP_HPC) may offer greater benefit by providing a higher concentration of growth factors to support tissue repair and regeneration. While our findings provide evidence-based guidance for protocol selection, clinical implementation should also consider feasibility factors such as preparation complexity, standardization, cost, and patient-specific characteristics. Further high-quality randomized trials with standardized PRP classification and longer follow-up durations are needed to strengthen the evidence base and optimize individualized treatment strategies.

## Electronic supplementary material

Below is the link to the electronic supplementary material.

Supplementary Material 1

Supplementary Material 2

Supplementary Material 3

Supplementary Material 4

Supplementary Material 5

## Data Availability

No datasets were generated or analysed during the current study.
