# Supplementary material for: The efficacy of platelet-rich plasma preparation protocols in the treatment of osteoarthritis: a network meta-analysis of randomized controlled trials
Source: J Orthop Surg Res. 2025 Jun 24;20:614. doi: 10.1186/s13018-025-06026-1 (PMC12186406; doi:10.1186/s13018-025-06026-1)
Supplement: Supplementary file 1 — Supplementary Material 1 [file 13018_2025_6026_MOESM1_ESM.docx]

**Supplementary Data**

**Table S1. Search Strategy for Each Database**

| **Database** | **#** | **Search strategy** | **Results** |
| --- | --- | --- | --- |
| PubMed | 1 | “Osteoarthritis” [Mesh] | 78,157 |
|  | 2 | "osteoarthr*"[Title/Abstract] OR "OA"[Title/Abstract] | 113,052 |
|  | 3 | "degenerative"[Title/Abstract] AND ("arthritis"[Title/Abstract] OR "joint disease"[Title/Abstract]) | 6,730 |
|  | 4 | #1 OR #2 OR #3 | 137,568 |
|  | 5 | "platelet rich plasma"[MeSH] OR "blood transfusion"[MeSH] | 99,011 |
|  | 6 | “platelet rich” [Title/Abstract] AND (plasma[Title/Abstract]  OR therap*[Title/Abstract] OR fibrin[Title/Abstract]) | 15,903 |
|  | 7 | "platelet"[Title/Abstract] AND ("gel"[Title/Abstract] OR "concentrate"[Title/Abstract]) | 6,312 |
|  | 8 | "buffy layer"[Title/Abstract] OR "PRP"[Title/Abstract] OR "PRF"[Title/Abstract] | 24,468 |
|  | 9 | #5 OR #6 OR #7 OR #8 | 127,670 |
|  | 10 | (randomized controlled trial[pt] OR controlled clinical trial[pt] OR randomized[tiab] OR placebo[tiab] OR clinical trials as topic[mesh:noexp] OR randomly[tiab] OR trial[ti]) NOT (animals [mh] NOT (humans [mh] AND animals[mh])) | 1,444,109 |
|  | 11 | #4 AND #9 AND #10 | 519 |
|  | 1 | MeSH descriptor: [Osteoarthritis] explode all trees | 10,596 |
|  | 2 | (osteoarthr*):ti,ab,kw OR (OA):ti,ab,kw OR (degenerative AND (arthritis OR joint disease)):ti,ab,kw | 24,777 |
|  | 3 | #1 OR #2 | 24,777 |
| Cochrane library | 4 | MeSH descriptor: [Platelet-Rich Plasma] explode all trees | 964 |
|  | 5 | MeSH descriptor: [Blood Transfusion] explode all trees | 4,931 |
|  | 6 | (“platelet rich”):ti,ab,kw AND (plasma OR therap* OR fibrin):ti,ab,kw | 4,050 |
|  | 7 | ("platelet"):ti,ab,kw AND ("gel" OR "concentrate"):ti,ab,kw | 1,110 |
|  | 8 | ("buffy layer" OR "PRP" OR "PRF"):ti,ab,kw | 4,708 |
|  | 9 | #4 OR #5 OR #6 OR #7 OR #8 | 11,188 |
|  | 10 | #3 AND #9 | 764 |
| EMBASE | 1 | 'osteoarthritis'/exp | 166,254 |
|  | 2 | osteoarthr*:ab,ti OR oa:ab,ti | 154,777 |
|  | 3 | 'degenerative':ab,ti AND ('arthritis':ab,ti OR 'joint disease':ab,ti) | 8,985 |
|  | 4 | #1 OR #2 OR #3 | 218,530 |
|  | 5 | 'thrombocyte rich plasma'/exp OR 'blood transfusion'/exp | 249,742 |
|  | 6 | 'platelet rich':ab,ti AND (plasma:ab,ti OR therap*:ab,ti OR fibrin:ab,ti) OR ('platelet':ab,ti AND ('gel':ab,ti OR 'concentrate':ab,ti)) OR 'buffy layer':ab,ti OR 'prp':ab,ti OR 'prf':ab,ti | 48,370 |
|  | 7 | #5 OR #6 | 281,049 |
|  | 8 | 'crossover procedure':de OR 'double-blind procedure':de OR 'randomized controlled trial':de OR 'single-blind procedure':de OR random*:de,ab,ti OR factorial*:de,ab,ti OR crossover*:de,ab,ti OR ((cross NEXT/1 over*):de,ab,ti) OR placebo*:de,ab,ti OR ((doubl* NEAR/1 blind*):de,ab,ti) OR ((singl* NEAR/1 blind*):de,ab,ti) OR assign*:de,ab,ti OR allocat*:de,ab,ti OR volunteer*:de,ab,ti | 3,213,228 |
|  | 9 | #4 AND #7 AND #8 | 1,108 |

**Search date:** October 16, 2023
