# Supplementary material for: The efficacy of platelet-rich plasma preparation protocols in the treatment of osteoarthritis: a network meta-analysis of randomized controlled trials
Source: J Orthop Surg Res. 2025 Jun 24;20:614. doi: 10.1186/s13018-025-06026-1 (PMC12186406; doi:10.1186/s13018-025-06026-1)

Appendix2: Network evidence plot of available comparisons and studies. The size of every nodes corresponds to the number of patients included in the interventions. The width of the edges is proportional to the number of trials for each comparison between interventions.


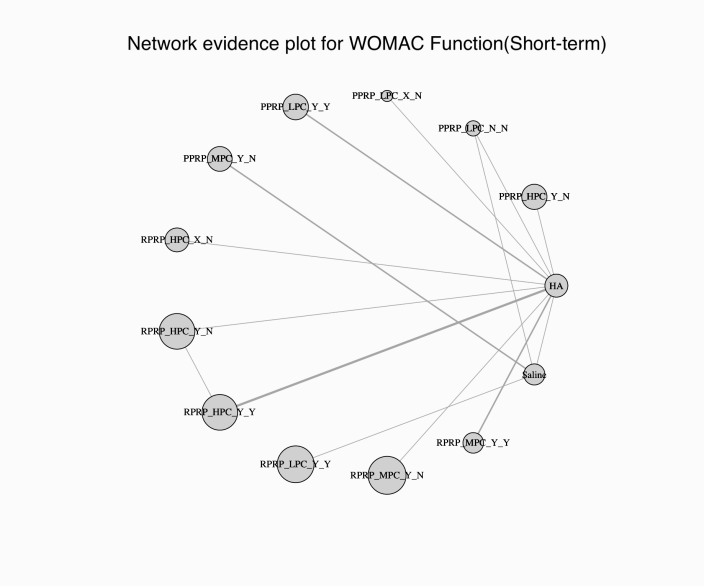

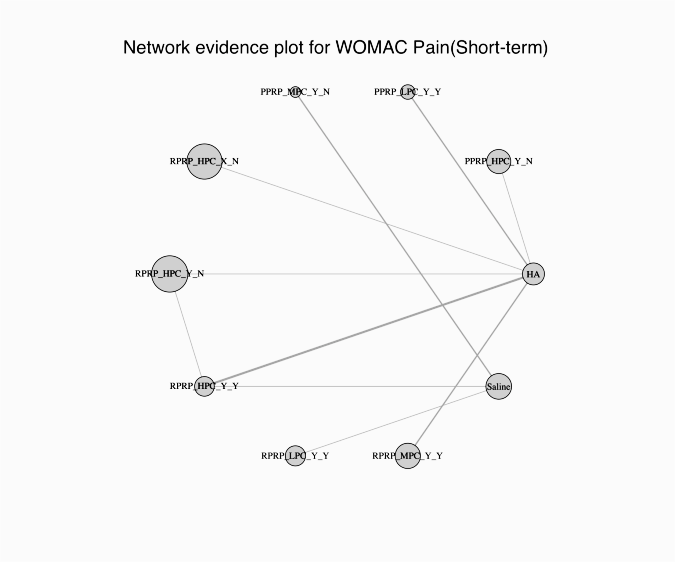


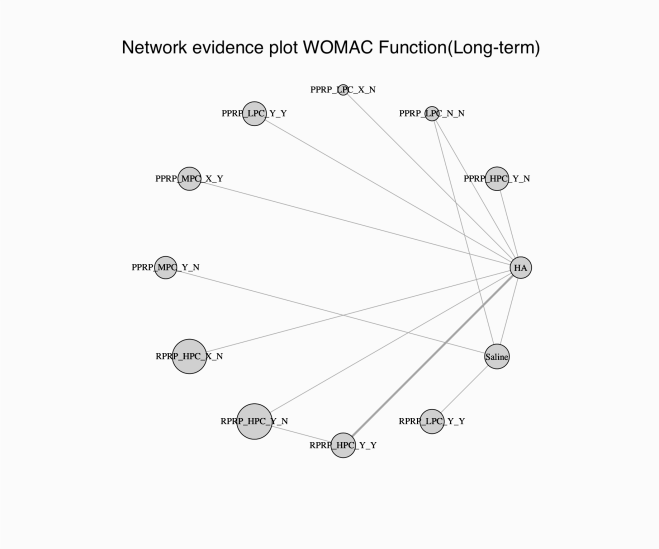

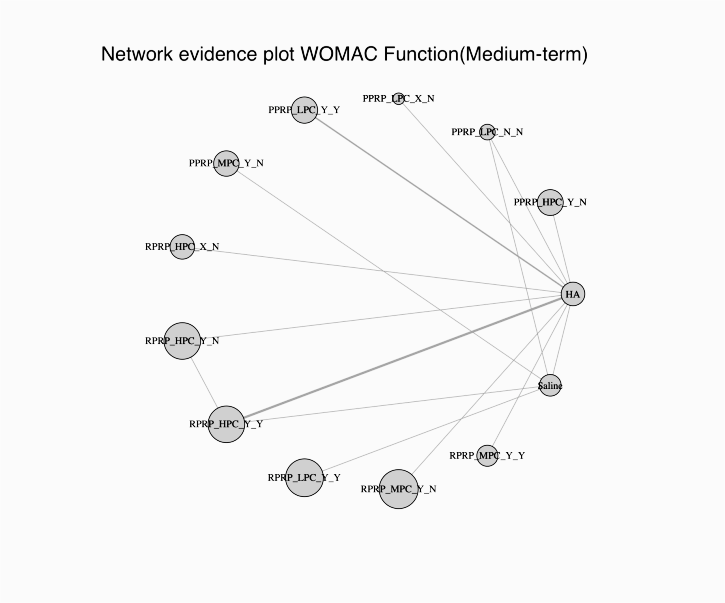

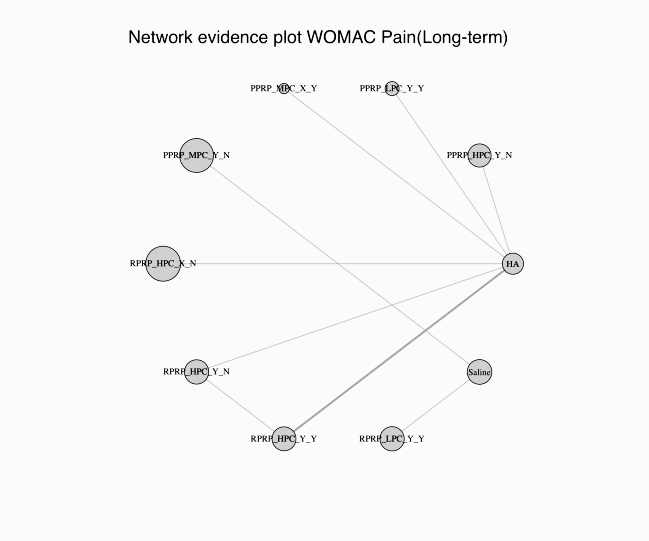

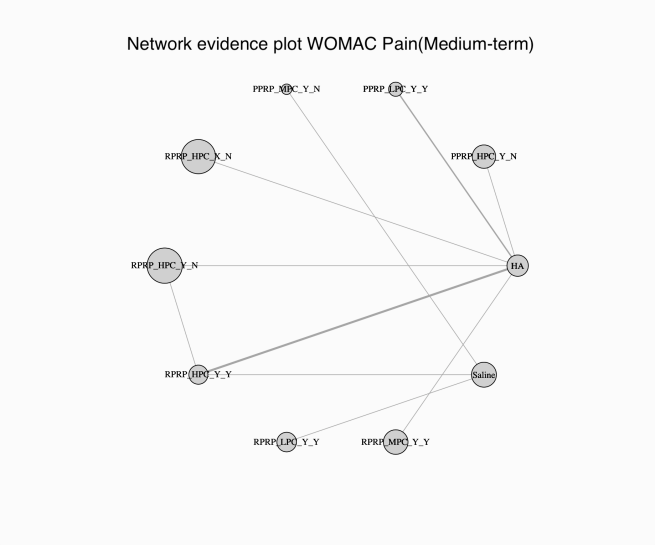


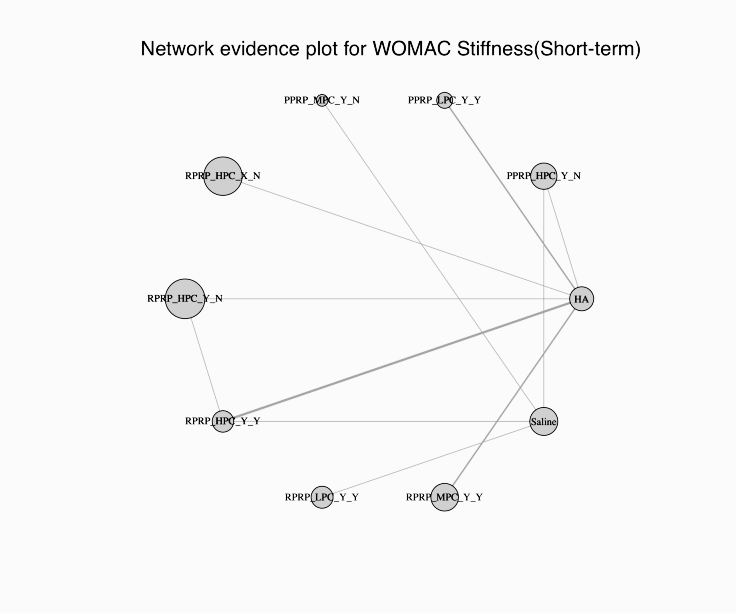

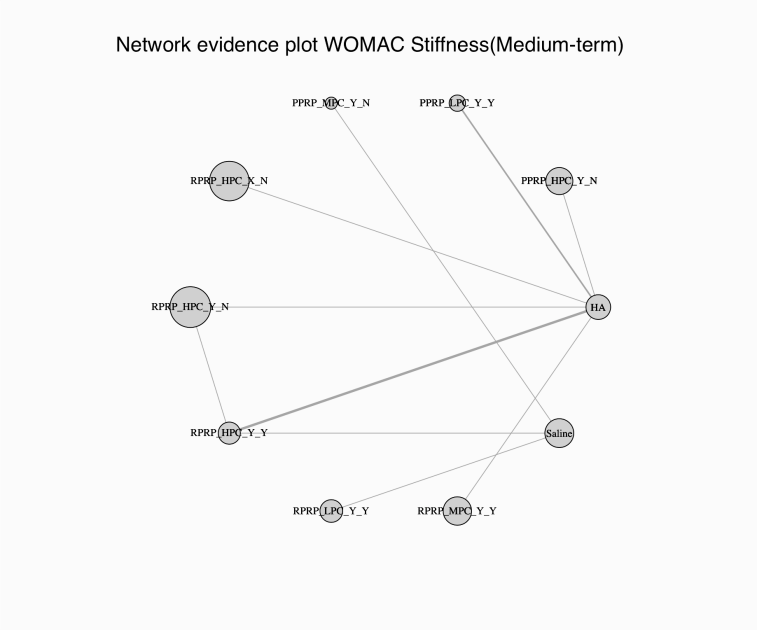

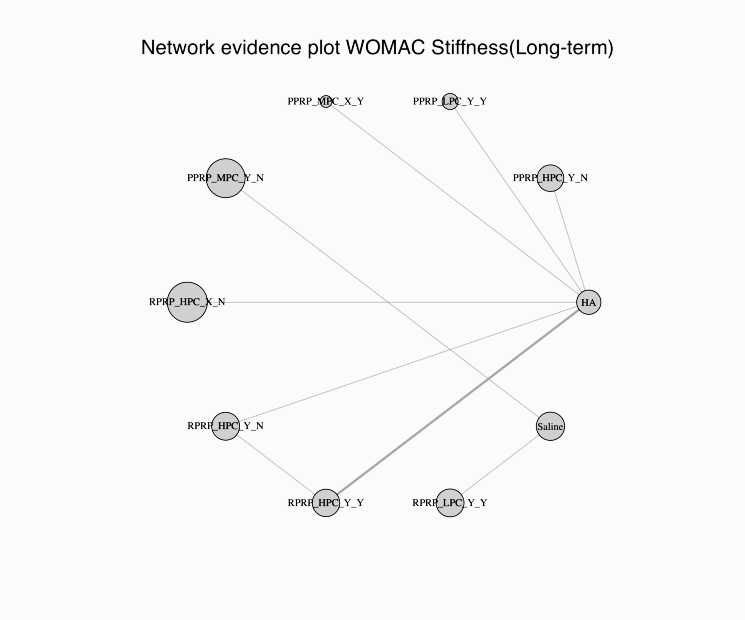

Supplement: Supplementary file 2 — Supplementary Material 2 [file 13018_2025_6026_MOESM2_ESM.docx]
