# Supplementary material for: The efficacy of platelet-rich plasma preparation protocols in the treatment of osteoarthritis: a network meta-analysis of randomized controlled trials
Source: J Orthop Surg Res. 2025 Jun 24;20:614. doi: 10.1186/s13018-025-06026-1 (PMC12186406; doi:10.1186/s13018-025-06026-1)

Appendix 3: Quality and Risk of Bias Assessment of the Included Studies

(Risk of bias summary: review authors’ judgments about each risk of bias item for each included study.)


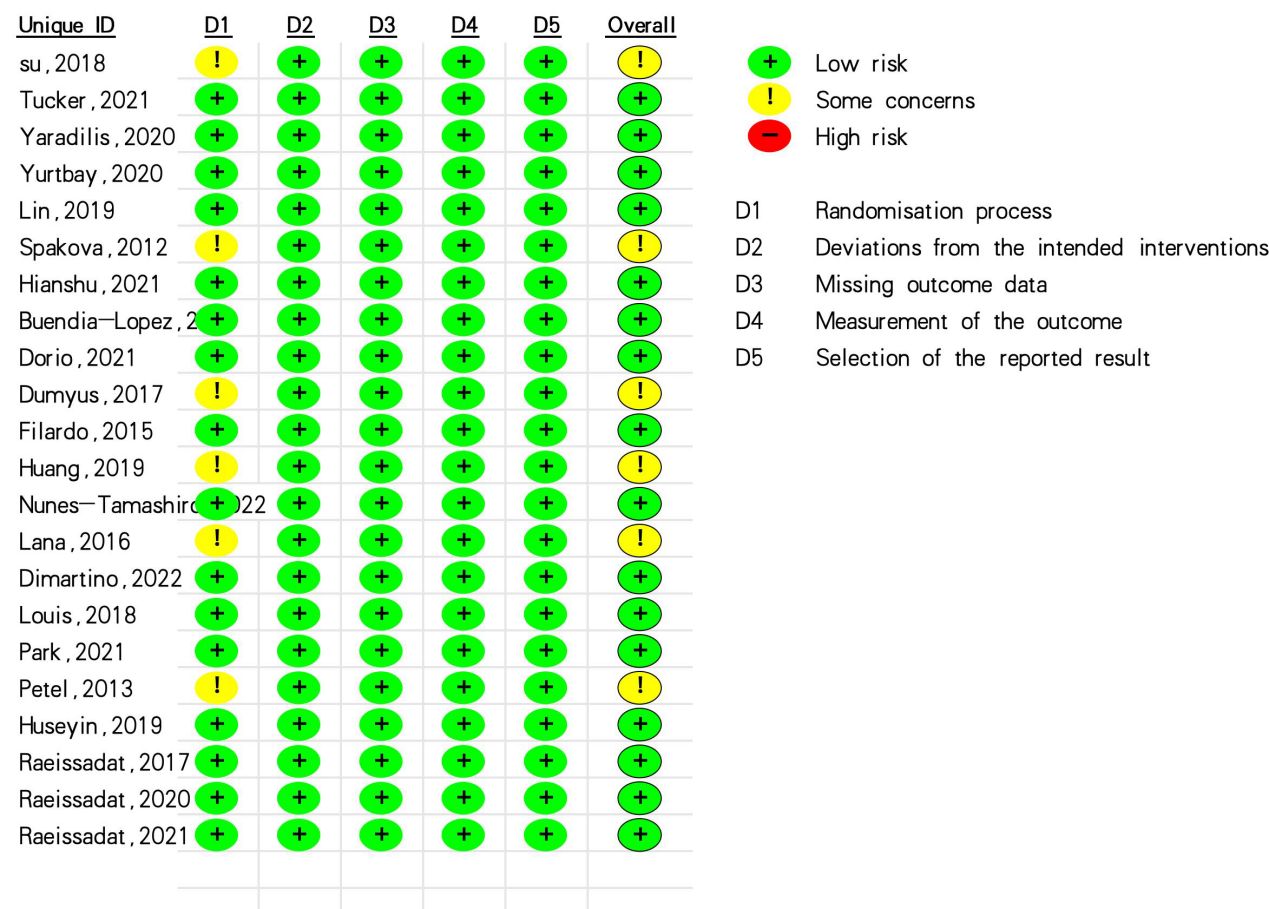


(Risk of bias summary: review authors’ judgments about each risk of bias item for each included study.)


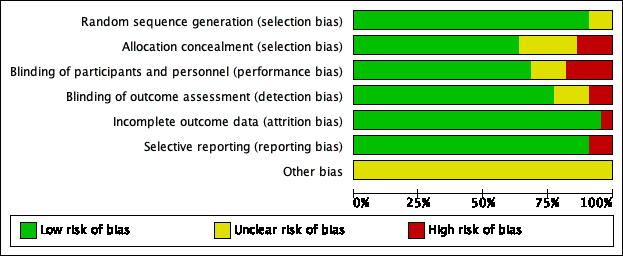

Supplement: Supplementary file 3 — Supplementary Material 3 [file 13018_2025_6026_MOESM3_ESM.docx]
