# Supplementary material for: The efficacy of platelet-rich plasma preparation protocols in the treatment of osteoarthritis: a network meta-analysis of randomized controlled trials
Source: J Orthop Surg Res. 2025 Jun 24;20:614. doi: 10.1186/s13018-025-06026-1 (PMC12186406; doi:10.1186/s13018-025-06026-1)
Supplement: Supplementary file 4 — Supplementary Material 4 [file 13018_2025_6026_MOESM4_ESM.docx]

Appendix 4: Results of Network Meta-analysis

Appendix Table 1.Network Meta-analysis Results of Short term WOMAC Pain Score

| HA |  |  | |  | |  | |  | |  | |  | |  | |  | |
| --- | --- | --- | --- | --- | --- | --- | --- | --- | --- | --- | --- | --- | --- | --- | --- | --- | --- |
| 4.27  (2.62, 5.91) | PPRP_HPC_Y_N | |  |  | |  | |  | |  | |  | |  | |  | |
| 0.48 (-0.77, 1.79) | -3.80 (-5.83, -1.67) | PPRP_LPC_Y_Y | | |  |  | |  | |  | |  | |  | |  | |
| 0.47 (-2.55, 3.56) | -3.80 (-7.24, -0.28) | 0.00  (-3.31, 3.32) | | PPRP_MPC_Y_N | | |  |  | |  | |  | |  | |  | |
| -1.44 (-3.11, 0.21) | -5.71  (-8.06, -3.37) | -1.92  (-4.04, 0.14) | | -1.91  (-5.46, 1.51) | | RPRP_HPC_X_N | | |  |  | |  | |  | |  | |
| 0.43 (-0.93, 1.93) | -3.84  (-5.95, -1.61) | -0.04 (-1.93, 1.88) | | -0.04 (-3.24, 3.17) | | 1.86  (-0.26, 4.14) | | RPRP_HPC_Y_N | | |  |  | |  | |  | |
| 0.32  (-0.53, 1.45) | -3.95  (-5.73, -1.86) | -0.15  (-1.67, 1.56) | | -0.14  (-3.01, 2.77) | | 1.77 (-0.06, 3.84) | | -0.12 (-1.46, 1.42) | | RPRP_HPC_Y_Y | | |  |  | |  | |
| 25.26 (3.23, 46.67) | 20.96  (-1.20, 42.41) | 24.73  (2.72, 46.10) | | 24.88  (2.37, 46.07) | | 26.74 (4.54, 48.11) | | 24.84  (2.75, 46.19) | | 24.96  (2.85, 46.30) | | RPRP_LPC_Y_Y | | |  |  | |
| 0.13 (-1.49, 1.84) | -4.14 (-6.42, -1.77) | -0.35  (-2.42, 1.76) | | -0.33  (-3.80, 3.16) | | 1.58  (-0.73, 3.99) | | -0.30 (-2.47, 1.88) | | -0.20  (-2.16, 1.69) | | -25.09 (-46.51, -2.93) | | RPRP_MPC_Y_Y | | |  |
| -1.15 (-3.72, 1.53) | -5.43 (-8.42, -2.25) | -1.63  (-4.51, 1.33) | | -1.62  (-3.19, -0.03) | | 0.27 (-2.74, 3.50) | | -1.59  (-4.35, 1.26) | | -1.48  (-3.91, 0.94) | | -26.42  (-47.70, -4.08) | | -1.26  (-4.40, 1.83) | | Saline | |

Appendix Table 2.Network Meta-analysis Results of Medium term WOMAC Pain Score

| HA |  |  | |  | |  | |  | |  | |  | |  | |  | |
| --- | --- | --- | --- | --- | --- | --- | --- | --- | --- | --- | --- | --- | --- | --- | --- | --- | --- |
| 3.33  (0.98, 5.71) | PPRP_HPC_Y_N | |  |  | |  | |  | |  | |  | |  | |  | |
| 0.94 (-0.80, 2.67) | -2.40  (-5.34, 0.61) | PPRP_LPC_Y_Y | | |  |  | |  | |  | |  | |  | |  | |
| -2.81  (-6.95, 1.58) | -6.15  (-10.79, -1.14) | -3.74  (-8.13, 1.03) | | PPRP_MPC_Y_N | | |  |  | |  | |  | |  | |  | |
| -0.90  (-3.20, 1.43) | -4.23  (-7.56, -0.90) | -1.82 (-4.70, 1.00) | | 1.90  (-3.03, 6.57) | | RPRP_HPC_X_N | | |  |  | |  | |  | |  | |
| 1.00  (-0.90, 3.24) | -2.33 (-5.33, 1.01) | 0.07  (-2.52, 2.97)" | | 3.83 (-0.72, 8.27) | | 1.90  (-1.05, 5.22) | | RPRP_HPC_Y_N | | |  |  | |  | |  | |
| 1.02  (-0.12, 2.68) | -2.30  (-4.83, 0.69)" | 0.10  (-1.88, 2.58) | | 3.85  (-0.13, 7.93) | | 1.91 (-0.54, 4.87) | | 0.02  (-1.99, 2.21) | | RPRP_HPC_Y_Y | | |  |  | |  | |
| 18.55 (-12.43, 46.90) | 15.17 (-16.12, 43.74) | 17.63 (-13.46, 46.07) | | 21.49  (-9.67, 49.32) | | 19.41  (-11.87, 47.73) | | 17.49 (-13.44, 46.01) | | 17.46  (-13.60, 45.73) | | RPRP_LPC_Y_Y | | |  |  | |
| 1.07  (-1.32, 3.53) | -2.24  (-5.63, 1.20) | 0.16  (-2.79, 3.15)" | | 3.90  (-1.11, 8.63) | | 1.99  (-1.37, 5.39) | | 0.07  (-3.27, 3.13)" | | 0.05  (-2.96, 2.64) | | -17.40 (-45.94, 14.01) | | RPRP_MPC_Y_Y | | |  |
| -2.15  (-5.12, 1.28) | -5.50  (-9.19, -1.28) | -3.08 (-6.46, 0.80) | | 0.65  (-2.14, 3.48) | | -1.24  (-4.94, 2.93)" | | -3.17  (-6.53, 0.47) | | -3.20  (-6.03, -0.29) | | -20.74  (-48.58, 10.31) | | -3.25  (-6.98, 0.95) | | Saline | |

Appendix Table 3.1.Network Meta-analysis Results of Long term WOMAC Pain Score

| HA |  |  | |  | |  | |  | |  | |
| --- | --- | --- | --- | --- | --- | --- | --- | --- | --- | --- | --- |
| 1.71  (-1.00, 4.44) | PPRP_HPC_Y_N | |  |  | |  | |  | |  | |
| 0.94  (-1.84, 3.71) | -0.78  (-4.67, 3.12) | PPRP_LPC_Y_Y | | |  |  | |  | |  | |
| 1.19  (-1.47, 3.85) | -0.53  (-4.33, 3.29) | 0.25 (-3.60, 4.10) | | PPRP_MPC_X_Y | | |  |  | |  | |
| 1.61 (-1.15, 4.31) | -0.12  (-3.97, 3.75) | 0.67  (-3.25, 4.55) | | 0.41  (-3.39, 4.21) | | RPRP_HPC_X_N | | |  |  | |
| 1.06  (-1.38, 3.54) | -0.66  (-4.27, 3.02) | 0.12  (-3.55, 3.87) | | -0.13  (-3.73, 3.53) | | -0.54  (-4.19, 3.19) | | RPRP_HPC_Y_N | | |  |
| 0.81  (-0.74, 2.52) | -0.91  (-4.00, 2.35) | -0.13 (-3.29, 3.17) | | -0.38 (-3.41, 2.82) | | -0.80  (-3.88, 2.48) | | -0.25  (-2.66, 2.28) | | RPRP_HPC_Y_Y | |

Appendix Table 4.2.Network Meta-analysis Results of Long term WOMAC Pain Score

| PPRP_MPC_Y_N |  |  |
| --- | --- | --- |
| 3.07 (-20.14, 26.63) | RPRP_LPC_Y_Y |  |
| -2.46  (-10.05, 5.09) | -5.48  (-27.97, 16.62) | Saline |

Appendix Table 4.Network Meta-analysis Results of Short term WOMAC Function Score

| HA |  |  |  |  |  |  |  |  |  |  |  |  |
| --- | --- | --- | --- | --- | --- | --- | --- | --- | --- | --- | --- | --- |
| 11.95  (6.62, 17.23) | PPRP_HPC_Y_N |  |  |  |  |  |  |  |  |  |  |  |
| -6.67  (-16.61, 3.12) | -18.62  (-29.95, -7.54) | PPRP_LPC_N_N |  |  |  |  |  |  |  |  |  |  |
| 0.85  (-4.55, 6.21) | -11.07  (-18.72, -3.51) | 7.50 (-3.57, 18.80) | PPRP_LPC_X_N |  |  |  |  |  |  |  |  |  |
| 3.25  (-1.01, 7.44) | -8.67  (-15.57, -1.88) | 9.96 (-0.78, 20.63) | 2.41  (-4.44, 9.24) | PPRP_LPC_Y_Y |  |  |  |  |  |  |  |  |
| 10.55  (-0.68, 21.72) | -1.41 (-13.91, 10.92) | 17.32  (6.12, 28.21) | 9.71  (-2.78, 22.10)) | 7.24 (-4.83, 19.39) | PPRP_MPC_Y_N |  |  |  |  |  |  |  |
| 3.24  (-2.60, 9.18) | -8.70  (-16.49, -0.79) | 9.97  (-1.45, 21.50) | 2.40 (-5.50, 10.41) | -0.01  (-7.11, 7.27) | -7.28  (-19.74, 5.51) | RPRP_HPC_X_N |  |  |  |  |  |  |
| 0.65  (-4.72, 5.38) | -11.28  (-18.96, -4.24) | 7.28 (-3.78, 18.29) | -0.18  (-7.82, 6.89) | -2.61  (-9.45, 3.60) | -9.93  (-22.23, 2.13) | -2.62  (-10.58, 4.80) | RPRP_HPC_Y_N |  |  |  |  |  |
| 1.92 (-1.88, 4.67) | -10.08  (-16.79, -4.29) | 8.42  (-2.02, 18.66) | 1.01  (-5.70, 6.88) | -1.41  (-7.19, 3.56) | -8.75 (-20.59, 2.71) | -1.39  (-8.54, 4.87) | 1.22 (-3.90, 6.06) | RPRP_HPC_Y_Y |  |  |  |  |
| 22.32  (-3.06, 47.45) | 10.42 (-15.68, 36.13) | 28.97  (3.50, 54.32) | 21.55  (-4.09, 47.23) | 19.14  (-6.87, 44.55) | 11.77 (-12.42, 35.79) | 19.15 (-6.72, 44.95) | 21.76  (-3.73, 47.46) | 20.63  (-5.17, 45.92) | RPRP_LPC_Y_Y |  |  |  |
| 7.37 (-0.09, 14.69) | -4.59 (-13.51, 4.42) | 14.00  (1.61, 26.45) | 6.47  (-2.60, 15.53) | 4.12  (-4.37, 12.55) | -3.19 (-16.58, 10.29) | 4.14  (-5.29, 13.34) | 6.77  (-2.00, 15.89) | 5.55 (-2.30, 13.86) | -15.05  (-40.99, 11.30) | RPRP_MPC_Y_N |  |  |
| 1.55  (-3.27, 6.83) | -10.39 (-17.36, -2.91) | 8.24  (-2.60, 19.40) | 0.67  (-6.31, 8.25) | -1.68  (-8.11, 5.14) | -9.01 (-21.01, 3.49) | -1.73 (-9.19, 6.20) | 0.89  (-5.66, 8.57) | -0.30  (-5.67, 6.35) | -20.69 (-46.30, 4.89) | -5.82  (-14.46, 3.30) | RPRP_MPC_Y_Y |  |
| 3.25  (-6.50, 13.10) | -8.73  (-19.86, 2.43) | 10.00  (0.27, 19.58) | 2.35 (-8.76, 13.56) | -0.13 (-10.69, 10.87) | -7.29  (-12.59, -1.85) | 0.02  (-11.47, 11.31) | 2.63  (-8.19, 13.76) | 1.45 (-8.57, 11.92) | -18.98 (-42.73, 4.47) | -4.13  (-16.37, 8.22) | 1.76 (-9.37, 12.50) | Saline |

Appendix Table 5.Network Meta-analysis Results of Medium term WOMAC Function Score

| HA |  |  |  |  |  |  |  |  |  |  |  |  |
| --- | --- | --- | --- | --- | --- | --- | --- | --- | --- | --- | --- | --- |
| 12.42  (5.76, 19.24) | PPRP_HPC_Y_N |  |  |  |  |  |  |  |  |  |  |  |
| -12.53 (-22.44, -2.11) | -24.95  (-36.47, -12.60) | PPRP_LPC_N_N |  |  |  |  |  |  |  |  |  |  |
| 6.23  (-0.65, 13.16) | -6.19 (-15.93, 3.42) | 18.82  (6.40, 30.17) | PPRP_LPC_X_N |  |  |  |  |  |  |  |  |  |
| 4.87  (-0.21, 10.20) | -7.55  (-15.93, 0.92) | 17.53  (5.81, 28.32) | -1.37  (-9.80, 7.41) | PPRP_LPC_Y_Y |  |  |  |  |  |  |  |  |
| -6.53  (-18.08, 6.33) | -18.96 (-32.09, -4.56) | 6.13  (-7.49, 19.61) | -12.71  (-25.83, 1.63) | -11.43  (-23.85, 2.29) | PPRP_MPC_Y_N |  |  |  |  |  |  |  |
| 0.69  (-6.24, 7.60) | -11.74  (-21.37, -2.25) | 13.24  (0.76, 24.87) | -5.57  (-15.22, 4.25) | -4.21  (-13.02, 4.35) | 7.23  (-7.33, 20.29) | RPRP_HPC_X_N |  |  |  |  |  |  |
| 2.17  (-3.46, 9.11) | -10.26 (-18.83, -0.57) | 14.79  (3.28, 26.25) | -4.08  (-12.76, 5.88) | -2.68 (-10.37, 5.97) | 8.79  (-4.75, 21.74) | 1.50  (-7.38, 11.55) | RPRP_HPC_Y_N |  |  |  |  |  |
| 2.93 (0.16, 8.21) | -9.44  (-16.12, -0.45) | 15.71  (5.53, 26.25) | -3.27  (-9.91, 5.93) | -1.81  (-7.41, 5.84) | 9.67  (-2.45, 21.68) | 2.35  (-4.52, 11.51) | 0.82  (-4.84, 7.75) | RPRP_HPC_Y_Y |  |  |  |  |
| 9.10 (-20.07, 40.94) | -3.34  (-33.37, 28.88) | 22.01  (-9.51, 54.46) | 2.94  (-26.90, 35.21) | 4.20 (-25.66, 36.71) | 15.52  (-15.08, 48.13) | 8.43 (-21.65, 40.29) | 6.76  (-22.90, 39.45) | 5.82  (-23.30, 37.90) | RPRP_LPC_Y_Y |  |  |  |
| 7.59  (-0.59, 15.95) | -4.82  (-15.37, 5.74) | 20.13  (6.95, 32.68) | 1.42  (-9.26, 12.03) | 2.71  (-7.08, 12.52) | 14.12  (-1.21, 28.23) | 6.93  (-3.86, 17.80) | 5.40  (-5.53, 15.22) | 4.51  (-5.61, 12.89) | -1.33  (-33.67, 28.82) | RPRP_MPC_Y_N |  |  |
| 4.25  (-3.19, 11.64) | -8.19  (-18.20, 1.73) | 16.81  (3.95, 28.71) | -1.96  (-12.15, 8.23) | -0.66  (-9.82, 8.35) | 10.74  (-4.13, 24.63) | 3.59  (-6.61, 13.54) | 2.07 (-8.23, 11.30) | 1.19  (-8.34, 8.61) | -4.85  (-37.42, 25.26) | -3.32  (-14.51, 7.61) | RPRP_MPC_Y_Y |  |
| -6.50  (-13.37, 1.53) | -18.94  (-28.12, -8.34) | 6.10  (-3.59, 15.70) | -12.77  (-21.93, -1.91) | -11.40  (-19.80, -1.77) | -0.01  (-9.70, 9.69) | -7.21 (-16.57, 3.61) | -8.76 (-17.54, 0.66) | -9.58  (-16.93, -2.44) | -15.62  (-46.75, 13.31) | -14.18  (-24.57, -2.41) | -10.79  (-20.68, 0.34) | Saline |

Appendix Table 6.Network Meta-analysis Results of Long term WOMAC Function Score

| HA |  |  |  |  |  |  |  |  |  |  |  |
| --- | --- | --- | --- | --- | --- | --- | --- | --- | --- | --- | --- |
| 4.18  (-12.86, 21.09) | PPRP_HPC_Y_N |  |  |  |  |  |  |  |  |  |  |
| -14.07  (-33.41, 5.09) | -18.23 (-44.01, 7.21) | PPRP_LPC_N_N |  |  |  |  |  |  |  |  |  |
| 15.48 (-1.44, 32.56) | 11.26  (-12.94, 35.45) | 29.48  (3.83, 55.42) | PPRP_LPC_X_N |  |  |  |  |  |  |  |  |
| 6.35 (-11.00, 23.19) | 2.15  (-22.01, 26.14) | 20.40  (-5.46, 45.99) | -9.12  (-33.52, 14.91) | PPRP_LPC_Y_Y |  |  |  |  |  |  |  |
| 6.27 (-11.04, 23.42) | 2.13 (-22.29, 26.11) | 20.31  (-5.34, 45.92) | -9.17  (-33.48, 15.25) | -0.06  (-24.32, 24.23) | PPRP_MPC_X_Y |  |  |  |  |  |  |
| 9.58 (-16.38, 35.05) | 5.44 (-25.50, 36.13) | 23.67  (-2.00, 49.08) | -5.88  (-36.71, 24.67) | 3.22  (-27.53, 34.43) | 3.35  (-27.75, 34.09) | PPRP_MPC_Y_N |  |  |  |  |  |
| 11.20  (-6.04, 27.96) | 6.97  (-17.10, 31.27) | 25.26  (-0.17, 50.84) | -4.25  (-28.50, 19.57) | 4.88  (-19.39, 29.09) | 4.96  (-19.36, 29.11) | 1.61 (-29.17, 32.24) | RPRP_HPC_X_N |  |  |  |  |
| 4.79 (-10.55, 20.79) | 0.55  (-22.12, 24.09) | 18.93 (-5.43, 43.92) | -10.66  (-33.42, 12.87) | -1.56  (-24.08, 22.32) | -1.45  (-24.22, 22.33) | -4.72  (-34.26, 25.94) | -6.40  (-28.92, 17.19) | RPRP_HPC_Y_N |  |  |  |
| 5.98  (-3.46, 16.63) | 1.69 (-17.10, 22.50) | 20.14 (-0.92, 42.39) | -9.51  (-28.63, 10.92) | -0.38  (-19.17, 20.41) | -0.33  (-19.54, 20.50) | -3.51  (-30.34, 25.11) | -5.31  (-24.12, 15.54) | 1.11  (-14.07, 17.02) | RPRP_HPC_Y_Y |  |  |
| -9.53 (-44.37, 24.61) | -13.75  (-52.22, 24.01) | 4.58  (-29.76, 38.76) | -24.93 (-63.49, 12.89) | -15.81  (-54.25, 22.27) | -15.84  (-53.91, 22.15) | -19.03  (-52.71, 14.38) | -20.66  (-59.00, 17.15) | -14.43  (-52.29, 22.51) | -15.73  (-52.22, 19.43) | RPRP_LPC_Y_Y |  |
| -1.60 (-20.73, 17.06) | -5.80  (-31.16, 19.31) | 12.46  (-6.41, 30.96) | -17.09  (-42.78, 8.40) | -7.96  (-33.46, 17.58) | -7.85  (-33.61, 17.58) | -11.19  (-28.69, 6.35) | -12.81 (-38.18, 12.55) | -6.51  (-31.55, 17.38) | -7.73  (-30.06, 13.02) | 7.93 (-21.07, 36.94) | Saline |

Appendix Table 7.Network Meta-analysis Results of Short term WOMAC Stiffness Score

| HA |  |  | |  | |  | |  | |  | |  | |  | |  | |
| --- | --- | --- | --- | --- | --- | --- | --- | --- | --- | --- | --- | --- | --- | --- | --- | --- | --- |
| 1.98  (-0.01, 3.93) | PPRP_HPC_Y_N | |  |  | |  | |  | |  | |  | |  | |  | |
| 0.12  (-1.52, 1.82) | -1.86  (-4.40, 0.78) | PPRP_LPC_Y_Y | | |  |  | |  | |  | |  | |  | |  | |
| 1.43  (-1.77, 4.72) | -0.55  (-3.70, 2.71) | 1.30  (-2.28, 5.01) | | PPRP_MPC_Y_N | | |  |  | |  | |  | |  | |  | |
| 0.31  (-2.02, 2.62) | -1.69  (-4.70, 1.40) | 0.18 (-2.74, 3.03) | | -1.12 (-5.16, 2.81) | | RPRP_HPC_X_N | | |  |  | |  | |  | |  | |
| 0.24  (-1.81, 2.37) | -1.75  (-4.45, 1.08) | 0.12 (-2.54, 2.78) | | -1.18  (-4.91, 2.52) | | -0.06  (-3.13, 3.05) | | RPRP_HPC_Y_N | | |  |  | |  | |  | |
| 0.73  (-0.48, 2.07) | -1.26 (-3.29, 0.92) | 0.60 (-1.48, 2.74) | | -0.70  (-3.86, 2.47) | | 0.42  (-2.18, 3.13) | | 0.48  (-1.57, 2.59) | | RPRP_HPC_Y_Y | | |  |  | |  | |
| 26.49 (1.14, 52.99) | 24.52  (-0.76, 51.01) | 26.36  (0.95, 52.94) | | 25.04  (-0.26, 51.64) | | 26.21 (0.65, 52.79) | | 26.24  (0.79, 52.83) | | 25.71  (0.48, 52.26) | | RPRP_LPC_Y_Y | | |  |  | |
| -0.05  (-1.75, 1.66) | -2.03  (-4.60, 0.60) | -0.17  (-2.60, 2.22) | | -1.48  (-5.19, 2.15) | | -0.35  (-3.23, 2.53) | | -0.29  (-3.01, 2.38) | | -0.77 (-2.95, 1.30) | | -26.55  (-53.13, -1.06) | | RPRP_MPC_Y_Y | | |  |
| 0.42  (-1.68, 2.61) | -1.56  (-3.57, 0.58) | 0.30  (-2.38, 3.06) | | -1.00  (-3.46, 1.42) | | 0.12 (-2.95, 3.31) | | 0.18 (-2.65, 2.99) | | -0.30  (-2.32, 1.70) | | -26.02  (-52.46, -0.92) | | 0.47  (-2.22, 3.22) | | Saline | |

Appendix Table 8.Network Meta-analysis Results of Medium term WOMAC Stiffness Score

| HA |  |  | |  | |  | |  | |  | |  | |  | |  | |
| --- | --- | --- | --- | --- | --- | --- | --- | --- | --- | --- | --- | --- | --- | --- | --- | --- | --- |
| 2.14  (0.65, 3.61) | PPRP_HPC_Y_N | |  |  | |  | |  | |  | |  | |  | |  | |
| 0.24  (-0.79, 1.35) | -1.91  (-3.65, -0.03) | PPRP_LPC_Y_Y | | |  |  | |  | |  | |  | |  | |  | |
| -1.19  (-3.73, 1.29) | -3.33  (-6.25, -0.49) | -1.44  (-4.23, 1.21) | | PPRP_MPC_Y_N | | |  |  | |  | |  | |  | |  | |
| 0.31  (-1.13, 1.75) | -1.83  (-3.89, 0.29) | 0.07 (-1.77, 1.81) | | 1.49  (-1.33, 4.42) | | RPRP_HPC_X_N | | |  |  | |  | |  | |  | |
| 0.08  (-1.25 1.38) | -2.05  (-4.04, -0.08) | -0.15  (-1.93, 1.48) | | 1.27  (-1.37, 4.01) | | -0.23  (-2.21, 1.70) | | RPRP_HPC_Y_N | | |  |  | |  | |  | |
| 0.20 (-0.70, 1.00) | -1.93  (-3.67, -0.28) | -0.03  (-1.50, 1.23) | | 1.39  (-0.97, 3.75) | | -0.10  (-1.82, 1.54) | | 0.12  (-1.23, 1.41) | | RPRP_HPC_Y_Y | | |  |  | |  | |
| 15.72 (-18.18, 45.30) | 13.61 (-20.32, 43.34) | 15.47 (-18.40, 45.09) | | 16.96 (-16.95, 46.58) | | 15.44 (-18.54, 45.02) | | 15.63 (-18.33, 45.17) | | 15.53 (-18.39, 45.00) | | RPRP_LPC_Y_Y | | |  |  | |
| 0.19  (-1.31, 1.70) | -1.95  (-4.04, 0.15) | -0.05 (-1.91, 1.75) | | 1.38 (-1.48, 4.36) | | -0.12 (-2.21, 2.02) | | 0.12  (-1.85, 2.15) | | -0.01  (-1.67, 1.79) | | -15.54 (-45.24, 18.48) | | RPRP_MPC_Y_Y | | |  |
| -1.39  (-3.30, 0.43) | -3.52  (-5.96, -1.22) | -1.63 (-3.85, 0.41) | | -0.20  (-1.91, 1.48) | | -1.69  (-4.11, 0.60) | | -1.47  (-3.67, 0.60) | | -1.59 (-3.25, 0.05) | | -17.04 (-46.53, 16.72) | | -1.57  (-4.04, 0.74) | | Saline | |

Appendix Table 9.1.Network Meta-analysis Results of Long term WOMAC Stiffness Score

| HA |  |  | |  | |  | |  | |  | |
| --- | --- | --- | --- | --- | --- | --- | --- | --- | --- | --- | --- |
| 0.66  (-0.53, 1.86) | PPRP_HPC_Y_N | |  |  | |  | |  | |  | |
| 0.09  (-1.14, 1.32) | -0.57  (-2.28, 1.14) | PPRP_LPC_Y_Y | | |  |  | |  | |  | |
| 0.64 (-0.55, 1.81) | -0.01  (-1.70, 1.66) | 0.55  (-1.17, 2.23) | | PPRP_MPC_X_Y | | |  |  | |  | |
| 0.80 (-0.39, 1.98) | 0.14 (-1.54, 1.81) | 0.71  (-0.99, 2.42) | | 0.16  (-1.51, 1.83) | | RPRP_HPC_X_N | | |  |  | |
| 0.25 (-0.78, 1.38) | -0.40 (-1.97, 1.25) | 0.16  (-1.42, 1.83) | | -0.39  (-1.94, 1.26) | | -0.55  (-2.09, 1.10) | | RPRP_HPC_Y_N | | |  |
| 0.29 (-0.34, 1.07) | -0.37 (-1.68, 1.09) | 0.20  (-1.14, 1.69) | | -0.36  (-1.63, 1.10) | | -0.51  (-1.81, 0.95) | | 0.03 (-1.01, 1.16) | | RPRP_HPC_Y_Y | |

Appendix Table 9.2.Network Meta-analysis Results of Long term WOMAC Stiffness Score

| PPRP_MPC_Y_N |  |  |
| --- | --- | --- |
| -6.89 (-32.75, 18.84) | RPRP_LPC_Y_Y |  |
| -1.62  (-8.53, 5.29) | 5.26 (-19.62, 30.35) | Saline |
