# Supplementary material for: The efficacy of platelet-rich plasma preparation protocols in the treatment of osteoarthritis: a network meta-analysis of randomized controlled trials
Source: J Orthop Surg Res. 2025 Jun 24;20:614. doi: 10.1186/s13018-025-06026-1 (PMC12186406; doi:10.1186/s13018-025-06026-1)
Supplement: Supplementary file 5 — Supplementary Material 5 [file 13018_2025_6026_MOESM5_ESM.docx]

Appendix 5：Results of Meta-regression

(Mean, SD, and 95% CrI for the interaction estimate (b) by meta-regression)

WOMAC Pain (Short-term)

| **Covariate** | **Mean** | **SD** | **95% CrI** |
| --- | --- | --- | --- |
| Sample size | 0.04 | 0.08 | (-0.13, 0.21) |
| BMI | -0.55 | 0.83 | (-2.26, 1.16) |
| Age | 0.02 | 0.23 | (-0.46, 0.50) |
| Female | -2.43 | 5.68 | (-14.21, 9.35) |

WOMAC Pain (Medium-term)

| **Covariate** | **Mean** | **SD** | **95% CrI** |
| --- | --- | --- | --- |
| Sample size | 0.04 | 0.08 | (-0.13, 0.21) |
| BMI | -0.55 | 0.83 | (-2.26, 1.16) |
| Age | 0.02 | 0.23 | (-0.46, 0.50) |
| Female | -2.43 | 5.68 | (-14.21, 9.35) |

WOMAC Pain (Long-term)

| **Covariate** | **Mean** | **SD** | **95% CrI** |
| --- | --- | --- | --- |
| Sample size | 0.29 | 0.14 | (-0.01, 0.59) |
| BMI | -2.62 | 1.37 | (-5.56, 0.31) |
| Age | -0.08 | 0.42 | (-0.98, 0.82) |
| Female | 23.82 | 9.50 | (3.44, 44.20) |

WOMAC Function (Short-term)

| **Covariate** | **Mean** | **SD** | **95% CrI** |
| --- | --- | --- | --- |
| Sample size | 0.04 | 0.08 | (-0.13, 0.21) |
| BMI | -0.55 | 0.83 | (-2.26, 1.16) |
| Age | 0.02 | 0.23 | (-0.46, 0.50) |
| Female | -2.43 | 5.68 | (-14.21, 9.35) |

WOMAC Function (Medium-term)

| **Covariate** | **Mean** | **SD** | **95% CrI** |
| --- | --- | --- | --- |
| Sample size | 0.04 | 0.08 | (-0.13, 0.21) |
| BMI | -0.55 | 0.83 | (-2.26, 1.16) |
| Age | 0.02 | 0.23 | (-0.46, 0.50) |
| Female | -2.43 | 5.68 | (-14.21, 9.35) |

WOMAC Function (Long-term)

| **Covariate** | **Mean** | **SD** | **95% CrI** |
| --- | --- | --- | --- |
| Sample size | 0.11 | 0.14 | (-0.24, 0.45) |
| BMI | -1.26 | 1.27 | (-3.91, 1.39) |
| Age | 0.34 | 0.54 | (-0.79, 1.47) |
| Female | -13.92 | 12.29 | (-39.65, 11.81) |

WOMAC Stiffness (Short-term)

| **Covariate** | **Mean** | **SD** | **95% CrI** |
| --- | --- | --- | --- |
| Sample size | 0.04 | 0.08 | (-0.13, 0.21) |
| BMI | -0.55 | 0.83 | (-2.26, 1.16) |
| Age | 0.02 | 0.23 | (-0.46, 0.50) |
| Female | -2.43 | 5.68 | (-14.21, 9.35) |

WOMAC Stiffness (Medium-term)

| **Covariate** | **Mean** | **SD** | **95% CrI** |
| --- | --- | --- | --- |
| Sample size | 0.04 | 0.08 | (-0.13, 0.21) |
| BMI | -0.55 | 0.83 | (-2.26, 1.16) |
| Age | 0.02 | 0.23 | (-0.46, 0.50) |
| Female | -2.43 | 5.68 | (-14.21, 9.35) |

WOMAC Stiffness (Long-term)

| **Covariate** | **Mean** | **SD** | **95% CrI** |
| --- | --- | --- | --- |
| Sample size | 0.29 | 0.14 | (-0.01, 0.59) |
| BMI | -2.84 | 1.22 | (-5.45, -0.23) |
| Age | -0.23 | 0.37 | (-1.03, 0.57) |
| Female | 29.58 | 8.46 | (11.45, 47.72) |
